# IL-4R**α** signaling in CD4^+^CD25^+^FoxP3^+^ T regulatory cells restrains airway inflammation via limiting local tissue IL-33

**DOI:** 10.1172/jci.insight.136206

**Published:** 2020-10-15

**Authors:** Jermaine Khumalo, Frank Kirstein, Sabelo Hadebe, Frank Brombacher

**Affiliations:** 1 Division of Immunology, and South African Medical Research Council (SAMRC) Immunology of Infectious Diseases, Department of Pathology,; 2 International Centre for Genetic Engineering and Biotechnology (ICGEB), and; 3 Wellcome Centre for Infectious Diseases Research in Africa (CIDRI-Africa), Institute of Infectious Diseases and Molecular Medicine (IDM), Faculty of Health Sciences, University of Cape Town, Cape Town, South Africa.

**Keywords:** Immunology, Allergy

## Abstract

Impaired tolerance to innocuous particles during allergic asthma has been linked to increased plasticity of FoxP3^+^ regulatory T cells (Tregs) reprogramming into pathogenic effector cells, thus exacerbating airway disease. However, failure of tolerance mechanisms is driven by Th2 inflammatory signals. Therefore, the in vivo role of canonical IL-4 receptor α (IL-4Rα) signaling, an essential driver of Th2-type airway responses to allergens, on the regulatory function of FoxP3^+^ Tregs in allergic asthma was explored. Here, we used transgenic Foxp3^cre^ IL-4Rα^–/lox^ and littermate control mice to investigate the role of IL-4 and IL-13 signaling via Tregs in house dust mite–induced (HDM-induced) allergic airway disease. We sensitized mice intratracheally on day 0, challenged them on days 6–10, and analyzed airway hyperresponsiveness (AHR), airway inflammation, mucus production, and cellular profile on day 14. In the absence of IL-4Rα responsiveness on FoxP3^+^ Tregs, exacerbated AHR and airway inflammation were shown in HDM-sensitized mice. Interestingly, reduced induction of FoxP3^+^ Tregs accompanied increased IL-33 alarmin production and type 2 innate lymphoid cell activation in the lung, exacerbating airway hyperreactivity and lung eosinophilia. Taken together, our findings indicate that IL-4Rα–unresponsive FoxP3^+^ Tregs result in exaggerated innate Th2-type, IL-33–dependent airway inflammation and a break in tolerance during allergic asthma.

## Abstract 2

IL-4Rα signaling in Tregs suppresses cytokine secretion in type 2 innate lymphoid cells via competition for IL-33, IL-10 secretion, and reparative macrophage activation.

## Graphical abstract

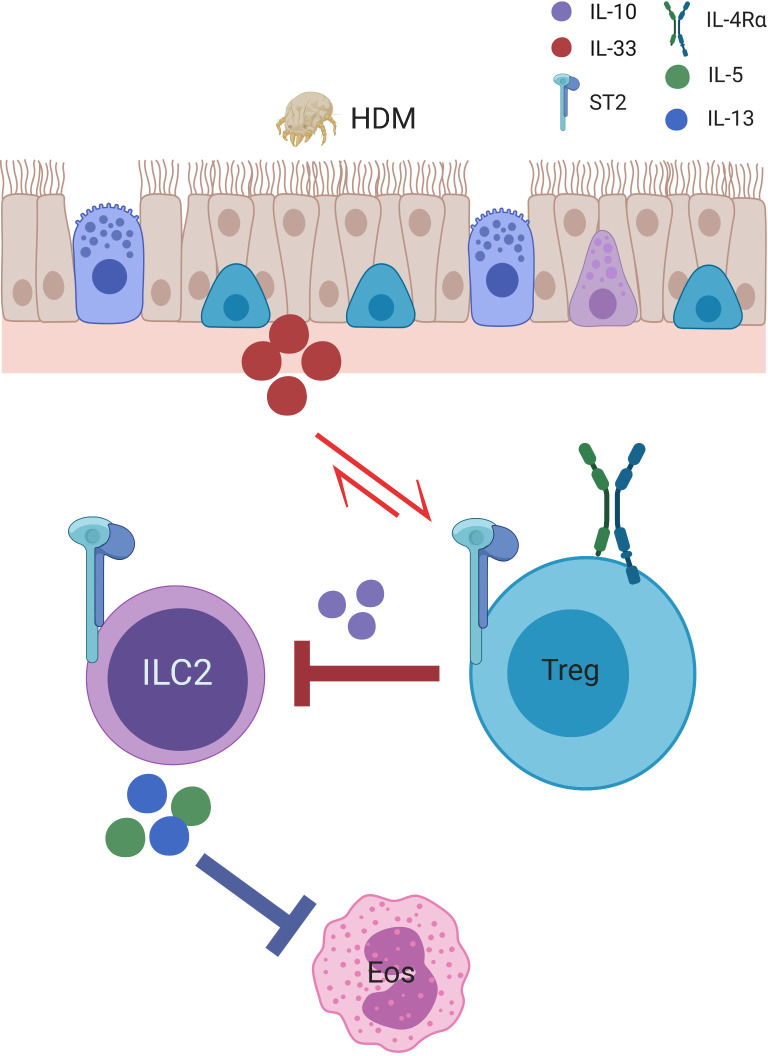

## Introduction

Allergic asthma is a chronic dysregulated airway immune response to harmless ubiquitous environmental particles that includes many disease hallmarks, such as airway hyperresponsiveness (AHR), goblet cell hyperplasia, eosinophilia, allergen-specific IgE, and influx of effector Th2-associated cytokines (1). The disease is associated with failed tolerogenic mechanisms to ubiquitous allergens, leading to uncontrolled Th2 airway responses (2–4). Tolerance to allergens is induced by FoxP3^+^CD25^+^ Tregs (5). Moreover, mice and humans with dysfunctional FoxP3 present with neonatal development of severe allergies characterized by hyper mucosal Th2 cells (6, 7).

Tregs can induce tolerance in several ways and target a broad array of cells, including B and T cells, innate lymphoid cells (ILCs), and antigen-presenting cells (APCs), in diverse mechanisms that show complex specificity guided by local environmental cues and an inflammatory condition (8). These regulatory mechanisms include but are not limited to suppression of pathogenic effector T cells through secretion of immunosuppressive antiinflammatory IL-10 and TGF-β cytokines (4); consumption of IL-2 (9); cell-cell contact suppression with costimulatory receptors, cytotoxic T lymphocyte–associated protein 4 (CTLA-4) (10), inducible costimulatory molecule (ICOS) (11), and programmed death 1 (PD-1) (3); contact-dependent killing of APCs through perforin and granzyme B; or depletion of peptide/major histocompatibility complex (MHC) II from dendritic cells (12).

Tolerance to allergens at mucosal surfaces is induced by a specialized Treg subset, called induced Tregs (iTregs), which are extrathymically derived and exert their immunosuppressive function through IL-10 secretion (13). In allergic asthma, iTregs are generated de novo by specialized lung tissue–resident macrophages through secretion of TGF-β and retinal dehydrogenases (14). TGF-β is important in the generation of antigen-specific FoxP3^+^ Tregs, and its abrogation in vivo leads to uncontrolled Th2 allergic asthma (15). Although iTregs may play a major role in generation of tolerance to airborne allergens, additional tolerance mechanisms exist, including diversion of allergen-specific cells to non-Th2 cells, peripheral deletion of autoreactive cells to harmless allergens, and allergen ignorance (16, 17). The way in which Th2 cells break this tolerance and cause allergic asthma disease, however, is not yet well understood.

Th2 cells can impair induction of Tregs either by hijacking them to become effector Th2 cells or by limiting their ability to act as active Th2 suppressor cells (15). These mechanisms include reprogramming of Tregs toward a pathogenic effector T cell phenotype leading to unrestrained Th2 airway inflammatory response (18–22). This paradigm of a Th2-dependent impairment of Treg function suggests a requirement for IL-4 and its receptor signaling on Tregs in allergic asthma. Mechanistically, recent studies have shown that a mutation in IL-4Rα at position 709 (IL-4Rα^F709^), in which tyrosine is substituted for phenylalanine, leads to increased STAT6 activity and exaggerated Th2 mucosal responses and food allergy (20). This mutation is in the tyrosine inhibitory motif and causes unrestrained STAT6 phosphorylation. This phenotype in murine models phenocopies that observed in human IL-4R with hyper-STAT6 activation (23). Another human polymorphism in IL-4Rα, in which glutamine is substituted for arginine at position 576 (IL-4Rα^Q576R^), is associated with asthma exacerbations. Mice carrying a homozygous allele of Q576R show increased Th2 and Th17 airway responses to house dust mite (HDM) and impaired iTreg induction (24). These studies suggest that IL-4 and IL-4Rα signaling via downstream transcription factor STAT6 disrupt the differentiation of CD4^+^ T cells into FoxP3^+^ Tregs in vitro and in vivo (25). We and others have previously shown that deletion of IL-4Rα in iTregs disrupts Foxp3 stability, promoting an increased Th2 immunity and clearance of *Heligmosomoides polygyrus* (26) or susceptibility to *Schistosoma mansoni* (27).

Previous studies point to mechanisms in which IL-4, through its receptor, acts on FoxP3^+^ iTregs inducing their differentiation and reprogramming them into pathogenic ex-FoxP3^+^ Th2 or ex-FoxP3^+^ Th17 (19, 24, 26, 28). Other studies have shown that overexpression of Th2-associated transcriptional factors such as interferon regulatory factor 4 or GATA binding protein 3 (Gata3) specifically on Tregs promotes their transdifferentiation into effector Th2 cells (29). Deletion of these transcriptional factors prunes the ability of iTregs to suppress Th2 immune responses (30). This highlights the role of a Th2 environment in impairing Treg tolerance in allergic disease. However, the functional role of IL-4Rα signaling on FoxP3^+^ Tregs during allergic disease is not yet completely clear. In the present study, we show that deletion of IL-4Rα specifically on FoxP3^+^ Tregs results in unrestrained lung AHR and allergic airway disease, owing to reduced suppressive function of Tregs on type 2 innate lymphoid cells (ILC2s).

## Results

### IL-4Rα signaling on Tregs modulates expansion and the functional stability of CD4^+^CD25^+^FoxP3^+^ Tregs in vivo during allergic disease.

**Figure 1:**
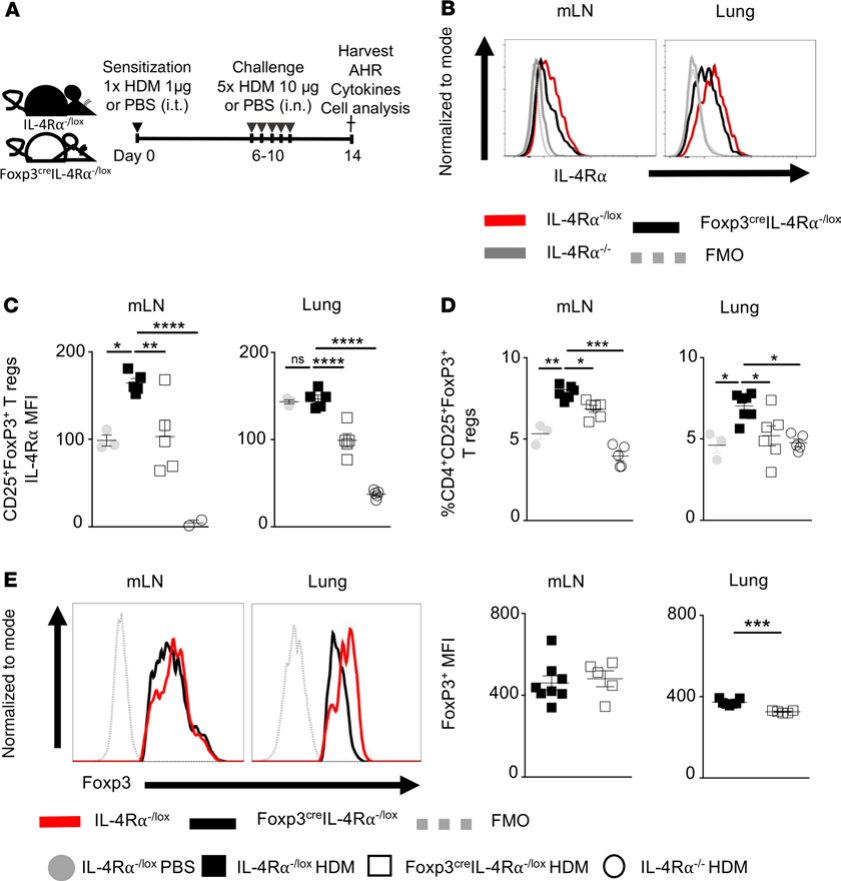
IL-4Rα–unresponsive CD4^+^CD25^+^FoxP3^+^ Tregs result in impaired Treg function mainly in the lung. (**A**) Schematic of the HDM-induced airway inflammation model, in which Foxp3^cre^ IL-4Rα^−/lox^ and IL-4Rα^−/lox^ mice were sensitized with HDM (1 μg) at day 0 intratracheally and challenged with HDM (10 μg) at days 6–11. AHR and FACS analyses were performed at day 14. (**B**) Flow cytometry histograms of Treg (CD3^+^CD4^+^CD25^+^FoxP3^+^) IL-4Rα expression in mediastinal lymph nodes (mLNs) and lung tissues. (**C**) Quantification of Treg (CD3^+^CD4^+^CD25^+^FoxP3^+^) IL-4Rα expression in mLN and lung tissue represented as MFI. Scatter plot represents mean ± SD from 1 representative experiment of 3 independent experiments (IL-4Rα^−/lox^ PBS *n* = 3, IL-4Rα^−/lox^ HDM *n* = 6, Foxp3^cre^ IL-4Rα^−/lox^ HDM *n* = 6, IL-4Rα^−/−^ HDM *n* = 5). (**D**) Proportion of CD4^+^CD25^+^FoxP3^+^ Tregs in mLN and lung tissue. Scatter plot represents mean ± SD of 1 representative experiment from 3 independent experiments (IL-4Rα^−/lox^ PBS *n* = 3, IL-4Rα^−/lox^ HDM *n* = 6, Foxp3^cre^ IL-4Rα^−/lox^ HDM *n* = 6, IL-4Rα^−/−^ HDM *n* = 5). (**E**) Flow cytometry histograms of FoxP3 expression of CD4^+^CD25^+^FoxP3^+^ Tregs in mLN and lung tissue. Quantitative MFI mean ± SEMs are shown (IL-4Rα^−/lox^ *n* = 8, Foxp3^cre^ IL-4Rα^−/lox^ *n* = 5). *P < 0.05, **P < 0.01, ***P < 0.001, ****P < 0.0001. One-way ANOVA with Tukey’s multicomparison test was performed. AHR, airway hyperresponsiveness; FMO, fluorescence minus one; MFI, median fluorescence intensity; ns, not significant.

The Foxp3^cre^ IL-4Rα^–/lox^ mouse strain has been previously characterized on the BALB/c background by our laboratory (27) and shown to have an impaired IL-4Rα expression on CD4^+^CD25^+^FoxP3^+^ Tregs in both lung and mediastinal lymph node (mLN) tissue (27). Following induction of HDM-induced allergic inflammation (Figure 1A), we showed by flow cytometry (gating strategy, Supplemental Figure 1; supplemental material available online with this article; https://doi.org/10.1172/jci.insight.136206DS1) a similarly markedly reduced median fluorescence intensity (MFI) on the expression of IL-4Rα subunit on CD4^+^CD25^+^FoxP3^+^ Tregs in the lung and mLN tissue in Foxp3^cre^ IL-4Rα^–/lox^ mice compared with IL-4Rα^–/lox^ littermate control mice, supporting effective deletion of the receptor (Figure 1, B and C). IL-4Rα expression was upregulated in mLN Tregs in IL-4Rα^–/lox^ mice sensitized and challenged with HDM compared with saline-treated control mice (Figure 1C). This suggested that HDM allergic inflammation also induced IL-4Rα signaling on Tregs in mLN tissue. Interestingly, we did not observe a massive induction of IL-4Rα expression by lung Tregs upon HDM challenge, which may suggest different dynamics occur in secondary lymphoid tissues and sites of disease.

Deletion of IL-4Rα in Foxp3^cre^ IL-4Rα^–/lox^ mice resulted in significantly impaired expansion of CD4^+^CD25^+^Foxp3^+^ Tregs in both mLN tissue and lungs compared with littermate control mice (Figure 1, D and E). Although the Foxp3 gene is not required for development of Tregs, it is necessary for their maintenance and stability (2, 31). We observed similar expression levels in FoxP3 in mLN Tregs in Foxp3^cre^ IL-4Rα^–/lox^ mice compared with IL-4Rα^–/lox^ controls treated with HDM, indicating a maintained Treg stability in the lymphoid tissue (Figure 1E). Interestingly, there was a marked reduction in FoxP3 expression in Tregs in Foxp3^cre^ IL-4Rα^–/lox^ mice when compared with littermate control Tregs (Figure 1E), highlighting a possible Treg instability in the local tissue. These findings suggest that IL-4Rα signaling in Tregs regulates expansion of Tregs in periphery and results in impaired expansion and functionality in the local lung tissue during airway inflammation.

### IL-4Rα–responsive CD4^+^CD25^+^FoxP3^+^ Tregs are required to control AHR and mucus hyperplasia in HDM-induced airway inflammation.

**Figure 2:**
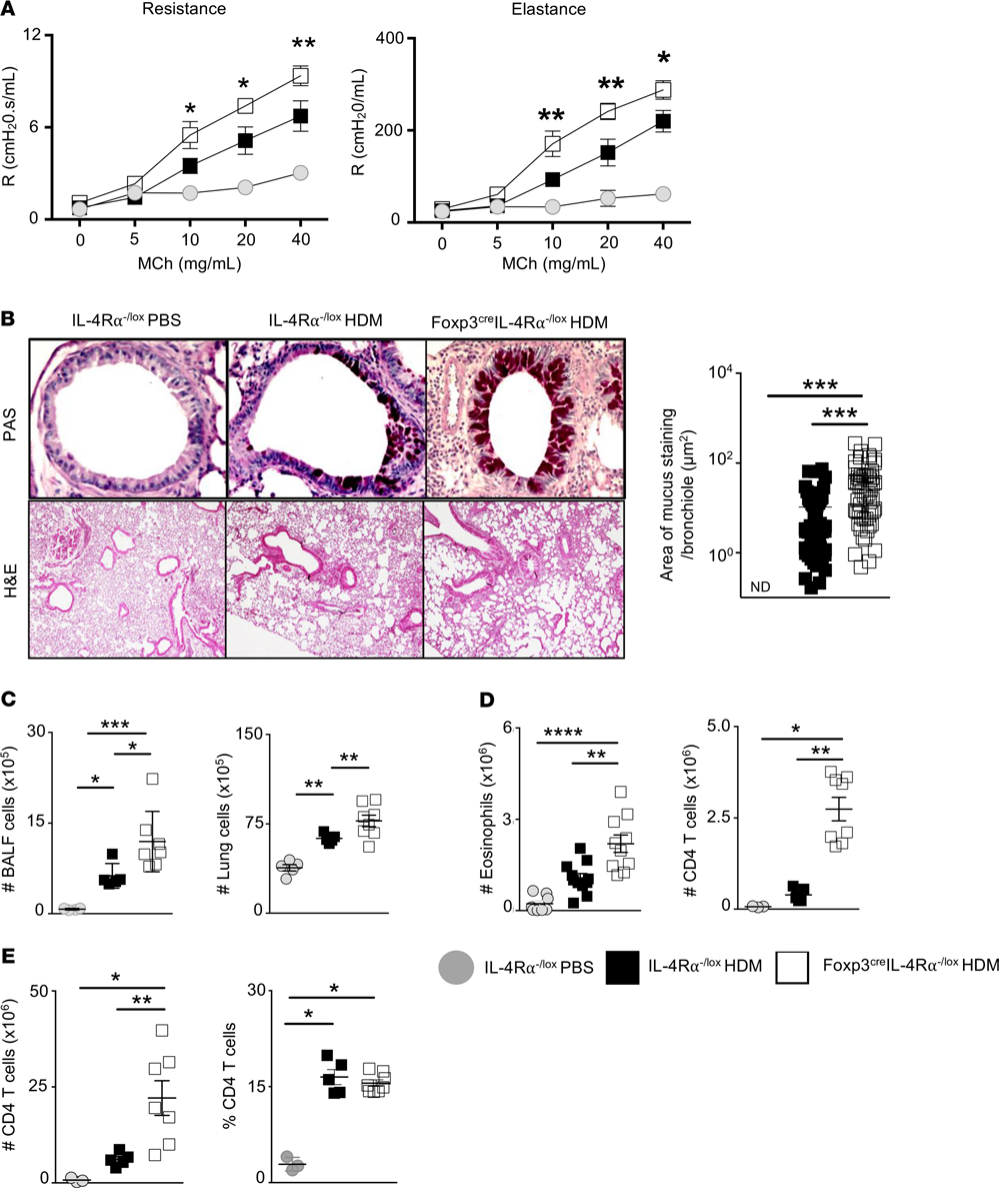
Deletion of IL-4Rα on CD4^+^CD25^+^FoxP3^+^ Tregs exacerbates AHR and mucus hyperplasia in HDM airway inflammation. (**A**) Airway resistance and airway elastance were measured using Flexivent by comparing dose-response curves to inhaled methacholine (0–40 mg/mL). Mean ± SEM is shown from 2 pooled experiments, including control saline mice (*n* = 7), Foxp3^cre^ IL-4Rα^−/lox^ mice (*n* = 10), and IL-4Rα^−/lox^ mice (*n* = 7). *P < 0.05, **P < 0.01. Two-way ANOVA with Bonferroni’s posttest was performed. (**B**) Histology of lung sections stained with periodic acid–Schiff (PAS) or H&E. Automated quantification of the area (μm^2^) of mucus staining per analyzed bronchiole was carried out using NIS Elements imaging software. PAS sections are magnified to ×200 and H&E slides are magnified to ×40. Scatter plots show mean ± SEMs (control saline mice *n* = 27, IL-4Rα^−/lox^ *n* = 57, Foxp3^cre^ IL-4Rα^−/lox^ *n* = 56). (**C**) Total cellular infiltration in bronchoalveolar lavage fluid and lung cells. (**D**) Number of lung eosinophils (live, CD11c^lo^CD11b^hi^Ly6G^lo^SiglecF^hi^) and CD4^+^ T cells (live, CD3^+^CD8^–^CD4^+^) analyzed by flow cytometry. (**E**) Number and frequencies of mLN CD4^+^ T cells (live, CD3^+^CD8^−^CD4^+^) analyzed by flow cytometry. (**C**–**E**) Scatter plots show mean ± SD of 1 representative experiment from 3 independent experiments (IL-4Rα^−/lox^ PBS *n* = 5, IL-4Rα^−/lox^ HDM *n* = 5, Foxp3^cre^ IL-4Rα^−/lox^ HDM *n* = 8). *P < 0.05, **P < 0.01, ***P < 0.001, **** *P* < 0.0001. One-way ANOVA with Tukey’s multicomparison test was performed.

STAT6 and IL-4 expression in vivo suppresses Tregs during allergic lung inflammation and is thought to dampen Treg suppressive function (32, 33). We next determined whether a lack of IL-4Rα signaling on Tregs would result in increased HDM-induced airway inflammation owing to reduced suppressive function. We found that HDM sensitization and challenge of Foxp3^cre^ IL-4Rα^–/lox^ mice resulted in significantly increased airway resistance and elastance compared with *IL-4Rα*^–/lox^** mice and PBS-treated control mice (Figure 2A). This result showed that deletion of the IL-4Rα on Tregs exacerbated AHR. We next measured mucus secretion in lung tissue sections and observed a significant increase in mucus production in Foxp3^cre^ IL-4Rα^–/lox^ mice compared with IL-4Rα^−/lox^ littermate control mice (Figure 2B). The increased airway obstruction correlated with modest increases in cellular infiltration in bronchoalveolar lavage (BAL) fluid and lung tissue of Foxp3^cre^ IL-4Rα^–/lox^ mice compared with IL-4Rα^–/lox^ littermate control mice (Figure 2C). We found similar cellular infiltration around the peribronchiolar and perivascular areas of H&E-stained lung tissue sections, with PBS control showing little inflammation (Figure 2B). Interestingly, eosinophil frequencies increased in lung tissue of Foxp3^cre^ IL-4Rα^–/lox^ mice compared with IL-4Rα^−/lox^ mice (Figure 2D). Moreover, we observed an accompanying increase in the proportions of CD4^+^ T cells in lung tissue of Foxp3^cre^ IL-4Rα^−/lox^ mice compared with IL-4Rα^–/lox^ littermate control mice, suggesting impaired TH cell suppression by Tregs (Figure 2D). The effector phenotype on TH cells was maintained along with the intrinsic IL-4Rα signaling in these cells (Supplemental Figure 2, A and B). Interestingly, no expansion of CD4^+^ T cells was observed in mLN tissue (Figure 2E), further suggesting a localized effect on suppressive function. These results clearly highlight an exacerbation in airway hyperreactivity, mucus secretion, and lung eosinophilia in IL-4 and IL-13 unresponsive Tregs.

**Figure 3:**
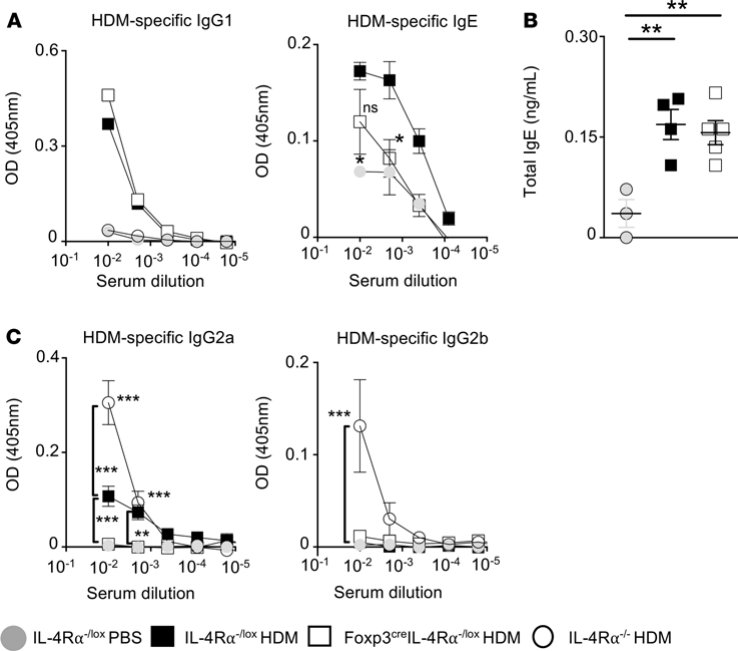
Deletion of IL-4Rα on CD4^+^CD25^+^FoxP3^+^ Tregs does not alter antibody production during HDM-induced allergic asthma. (**A**) HDM-specific IgG and HDM-specific IgE. (**B**) Total IgE. (**C**) HDM-specific IgG2a and HDM-specific IgG2b measured by ELISA. Mean ± SD are shown for 1 representative experiment from 3 independent experiments (IL-4Rα^−/lox^ PBS *n* = 3, IL-4Rα^−/lox^ HDM *n* = 5, Foxp3^cre^ IL-4Rα^−/lox^ *n* = 5). *P < 0.05, **P < 0.01, ***P < 0.001, ****P < 0.0001. One-way ANOVA with Tukey’s multicomparison test was performed.

We next measured serum levels of HDM-specific IgG1, IgG2a, and IgG2b, IgE, and total levels of IgE, and found similar levels of IgG1 and total IgE in Foxp3^cre^ IL-4Rα^–/lox^ mice and IL-4Rα^–/lox^ littermate control mice (Figure 3, A and B). Figure 3C shows a slight increase in HDM-specific IgG2b and IgG2a levels in IL-4Rα^–/lox^ mice compared with Foxp3^cre^ IL-4Rα^−/lox^ mice and PBS control mice and that these levels were significant in IgG2a. Figure 3A shows a modest reduction (i.e., not in all dilutions) in HDM-specific IgE production and a significant decrease in HDM-specific IgG2a in Foxp3^cre^ IL-4Rα^−/lox^ mice compared with IL-4Rα^–/lox^ mice. These results showed a persistent type 2 antibody production in the absence of IL-4Rα signaling on Tregs during HDM-induced allergic asthma.

### Altered Th2 immunity in lymphoid and local tissue of Foxp3^cre^ IL-4Rα^–/lox^ mice upon acute HDM challenge.

**Figure 4:**
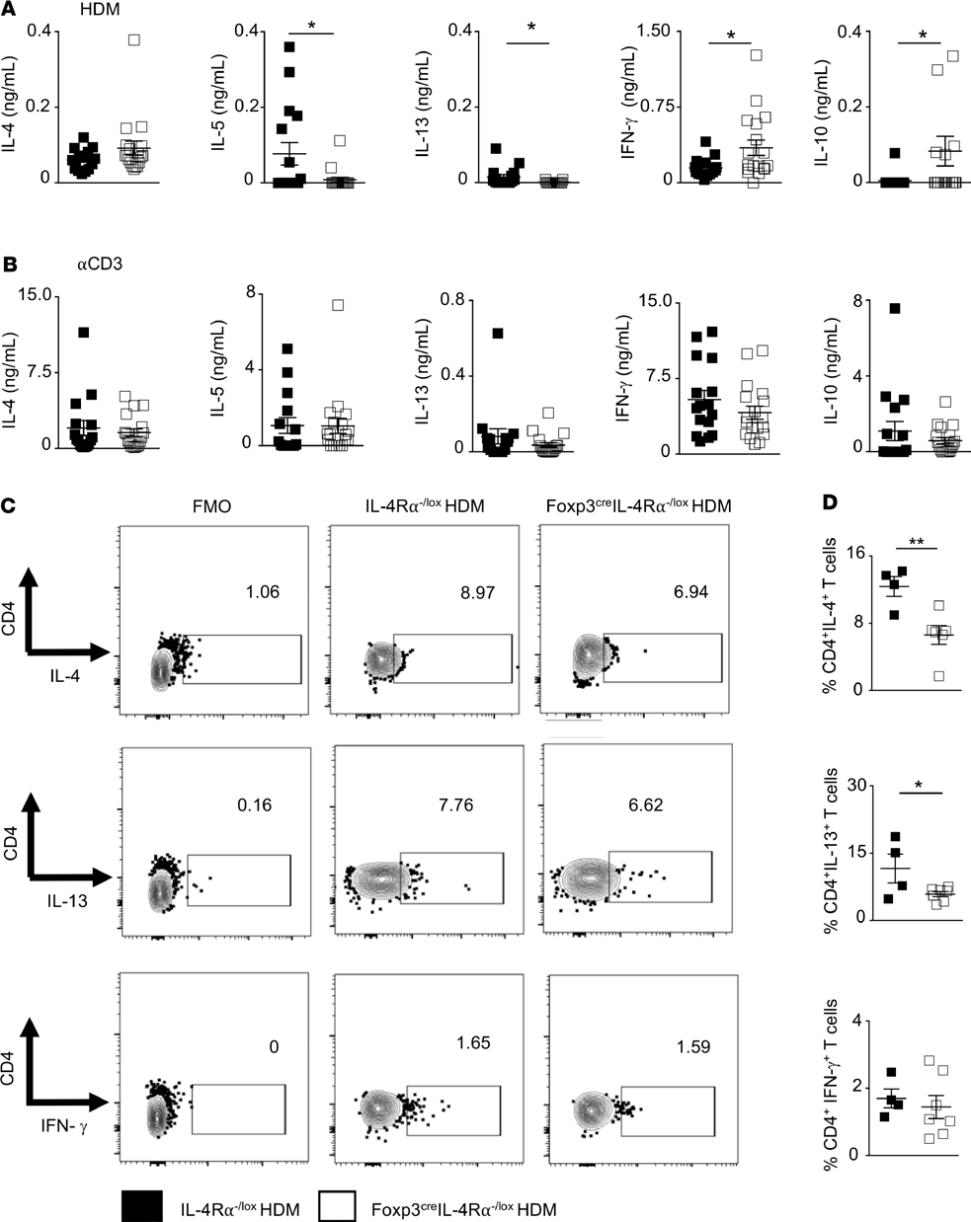
Impairment of HDM-specific Th2-associated cytokine production in peripheral lymphoid but not local lung tissue of FoxP3^cre^ IL-4Rα^−/lox^ mice during HDM-induced airway inflammation. Th2-associated cytokine production was measured in 5-day ex vivo restimulated mLNs using ELISA. (**A**) HDM-restimulated mLNs. Scatter plot represents mean ± SEM of 3 pooled experiments (IL-4Rα^−/lox^ *n* = 16, Foxp3^cre^ IL-4Rα^−/lox^ *n* = 17). (**B**) Anti-CD3–restimulated mLNs. Scatter plot represents mean ± SEM of 3 pooled experiments (IL-4Rα^−/lox^ *n* = 16, Foxp3^cre^ IL-4Rα^−/lox^ *n* = 17). (**C**) Flow cytometry plots for CD4^+^ T cells producing intracellular IL-4, IL-13, and IFN-γ after 5 hours’ stimulation with PMA/ionomycin and monensin. (**D**) Scatter plot frequencies of cytokine-producing CD4^+^ T cells based on percentages shown in **C**. Data represent mean ± SD from 1 representative experiment of 3 independent experiments (IL-4Rα^−/lox^ *n* = 4, Foxp3^cre^ IL-4Rα^−/lox^ *n* = 7). *P < 0.05. Mann-Whitney *U* test was performed.

IL-4 and IL-13 cytokines are crucial in the induction of AHR and mucus hyperplasia (34). Therefore, we measured Th2 cytokine production in both HDM- and anti-CD3–restimulated mLNs (Figure 4, A and B). Figure 4A shows a significant reduction in HDM-specific IL-5 and IL-13 and modest increase in IL-4 production in Foxp3^cre^ IL-4Rα^–/lox^ mice compared with IL-4Rα^–/lox^ littermate control mice. Similar findings were observed in anti-CD3–restimulated mLNs, particularly in IL-5 and IL-13; however, these levels did not reach statistical significance (Figure 4B). Regulatory HDM-specific IL-10 and Th1-associated IFN-γ cytokines increased in Foxp3^cre^ IL-4Rα^–/lox^ mice compared with IL-4Rα^–/lox^ littermate control mice. However, similar increases were not observed in these cytokines upon anti-CD3 restimulation (Figure 4B). Intracellular cytokine production was assessed in CD4^+^ T cells using flow cytometry in lungs (Figure 4, C and D; and Supplemental Figure 3) and mLNs (Supplemental Figure 3). Significant reduction was observed in frequencies of CD4^+^ T cells producing IL-4, IL-13, IL-10, and IL-17 (Figure 4, C and D; and Supplemental Figure 3). However, no changes were observed in total numbers of CD4^+^ T cells producing IL-4, IL-13, IL-10, IL-17, and IFN-γ (Supplemental Figure 3) in Foxp3^cre^ IL-4Rα^–/lox^ mice compared with IL-4Rα^–/lox^ littermate control mice. Similarly, Supplemental Figure 3 shows no significant changes in proportions and numbers of CD4^+^ T cells producing IL-4, IL-13, IL-10, IL-17, and IFN-γ. Our results show that in mLNs, Th2 cytokines may be suppressed by a strong induction of IL-10 and IFN-γ, whereas in the lung reduced Th2 induction may be independent of IL-10 and IFN-γ. Interestingly, despite these slight decreased frequencies in T cell–derived and Th2-associated cytokines (Figure 4, A–C), the disease pathology showed signs of increasing in the IL-4Rα–unresponsive Treg group. These data suggest there may be other regulators that contribute toward a significant increase in AHR and pathology and that these regulators may be tissue specific as recently reported (35).

### IL-4–responsive CD4^+^CD25^+^FoxP3^+^ Tregs regulate IL-33 production and ILC2 activation in the lung.

**Figure 5:**
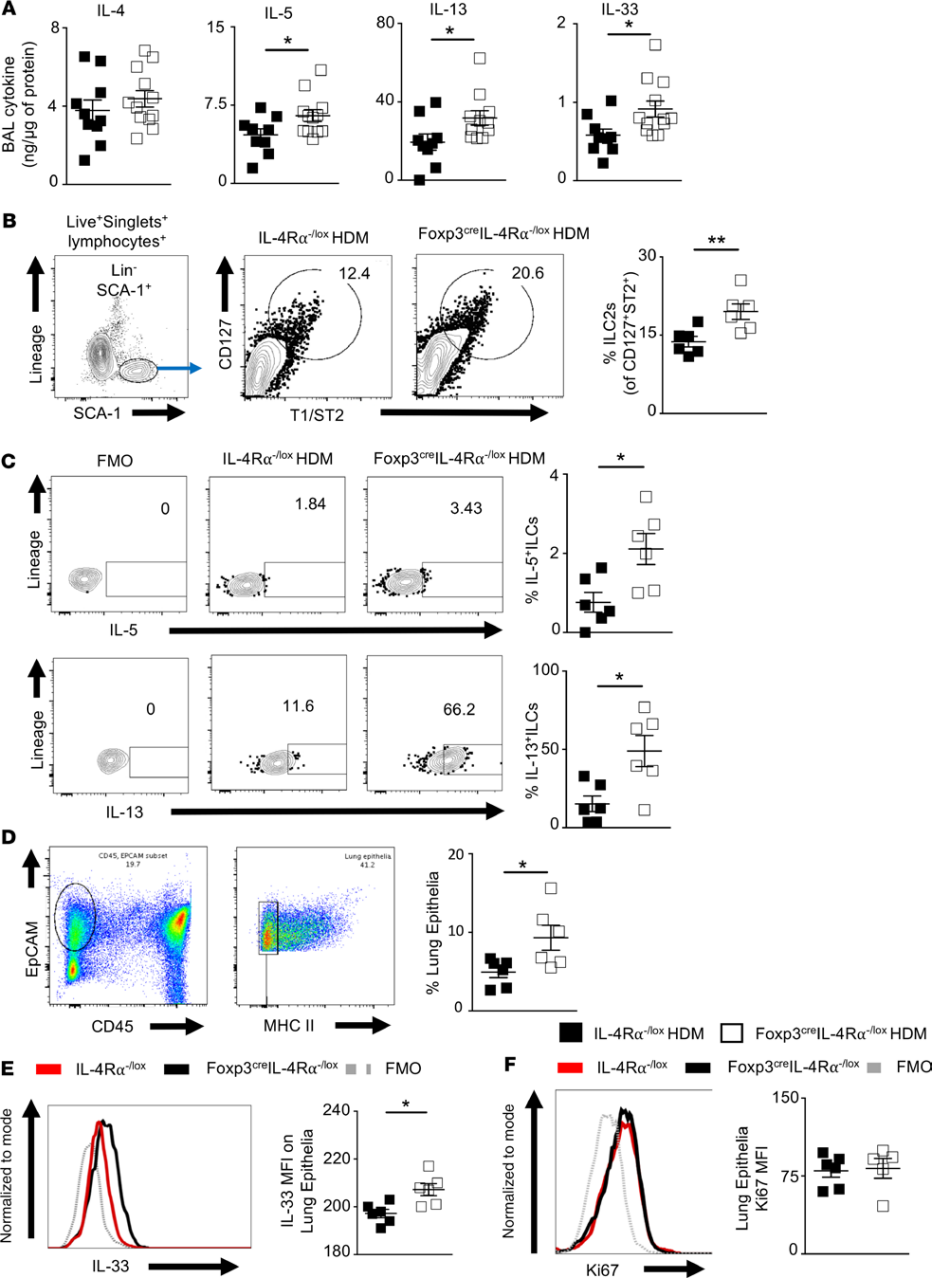
Exacerbated innate type 2 cytokine production in lung of Foxp3^cre^ IL-4Rα^−/lox^ mice upon acute HDM challenge. (**A**) BAL cytokines (IL-4, IL-5, IL-13, and IL-33) were measured by ELISA and corrected to the protein concentration. Scatter plot represents mean ± SEM of 2 pooled experiments (IL-4Rα^−/lox^ *n* = 10, Foxp3^cre^ IL-4Rα^−/lox^ *n* = 12). (**B**) Flow cytometry plots of ILC2s, live^+^ singlets, and lymphocyte lineage^−^ SCA^+^CD127^+^T1/ST2^+^ and quantification in frequency of ST2^+^CD127^+^. Scatter plot represents mean ± SD of 1 representative experiment from 2 independent experiments (IL-4Rα^−/lox^ *n* = 6, Foxp3^cre^ IL-4Rα^−/lox^ *n* = 6). (**C**) Flow cytometry plots and quantification of ILC2s producing intracellular IL-5 and IL-13 after 5 hours’ stimulation with PMA/ionomycin and monensin. Scatter plot data represent mean ± SD of 1 representative experiment from 2 independent experiments (IL-4Rα^−/lox^ *n* = 6, Foxp3^cre^ IL-4Rα^−/lox^ *n* = 6). (**D**) Flow cytometry plots and quantification of epithelial cells (live, CD45^–^EpCam^+^MHCII^–^). Scatter plot data represent mean ± SD of 1 representative experiment from 3 independent experiments (IL-4Rα^−/lox^ *n* = 6, Foxp3^cre^ IL-4Rα^−/lox^ *n* = 6). (**E**) Representative histogram plot and quantification of IL-33 MFI from epithelial cells measured by flow cytometry. Scatter plot data represent mean ± SD of 1 representative experiment from 2 independent experiments (IL-4Rα^−/lox^ *n* = 6, Foxp3^cre^ IL-4Rα^−/lox^ *n* = 6). (**F**) Flow cytometry histogram of Ki67 proliferative marker expression of epithelial cells as gated in **D** and MFI. Scatter plot data represent mean ± SD of 1 representative experiment from 2 independent experiments (IL-4Rα^−/lox^ *n* = 6, Foxp3^cre^ IL-4Rα^−/lox^ *n* = 6). *P < 0.05, **P < 0.01. Mann-Whitney *U* test was performed.

In the lung, ILC2s and natural killer T cells can secrete type 2 cytokines, causing pathology and AHR (36–38). Therefore, we next examined the BAL fluid and lung, which are sites of inflammation and pathology. We measured Th2 cytokine production in BAL fluid (Figure 5A). Similar levels of IL-4 in the BAL fluid were observed when comparing Foxp3^cre^ IL-4Rα^–/lox^ mice and IL-4Rα^−/lox^ littermate control mice (Figure 5A). Interestingly, we observed a significant increase in total IL-5 and IL-13 levels in BAL fluid, along with innate cytokine IL-33 in Foxp3^cre^ IL-4Rα^–/lox^ mice compared with IL-4Rα^−/lox^ littermate control mice (Figure 5A). These results suggested the local cytokine milieu may be the driver in increased pathology and that IL-33 may be a key mediator, as it is a key amplifier of mucosal and systemic innate responses (39).

ILC2s are recognized as the major producers of IL-5 and IL-13 in lung tissue and are mainly activated by IL-33 (37–40). Therefore, we next investigated this T cell–independent type 2 increase and the way in which IL-4Rα–responsive CD4^+^CD25^+^FoxP3^+^ Tregs could modulate it. In line with an increase in total IL-33 levels in the bronchoalveolar lavage fluid (BALF) (Figure 5A), a corresponding significant increase in ILC2 expansion was observed in Foxp3^cre^ IL-4Rα^–/lox^ mice compared with IL-4Rα^–/lox^ littermate control mice (Figure 5B). Conversely, the increase in ILC2s in the lung was complemented by an increase in ILC2s producing IL-5 and IL-13 intracellular cytokines in Foxp3^cre^ IL-4Rα^–/lox^ mice compared with IL-4Rα^–/lox^ littermate control mice (Figure 5C). This result highlights the regulatory role of IL-4Rα–responsive Tregs in local lung tissue inflammation, particularly in suppressing ILC2-derived type 2 inflammatory cytokines during HDM-induced allergic airway inflammation.

Epithelial cells secrete IL-33 as an alarmin to any barrier damage during airway inflammation, particularly by proteases (41). Figure 5D shows increased proportions of epithelial cells in Foxp3^cre^ IL-4Rα^–/lox^ mice when compared with littermate control IL-4Rα^–/lox^ mice (Figure 5D). Figure 5E shows increased IL-33 expression by lung epithelia cells in Foxp3^cre^ IL-4Rα^–/lox^ mice compared with IL-4Rα^–/lox^ mice littermate control, corroborating increased ILC2 induction. Interestingly, the increase in proportion of lung epithelia cells in IL-4Rα–deficient Tregs was not accompanied by an increase in proliferative capacity as measured by Ki67 staining but likely was attributed to an increase in goblet cells or general epithelial damage (Figure 5F and Figure 2B). Taken together, these results demonstrate the importance of IL-4Rα–responsive CD4^+^CD25^+^FoxP3^+^ Tregs in modulating innate cytokine production during acute lung inflammation.

### IL-4Rα signaling modulates IL-10 production in the lung CD4^+^CD25^+^FoxP3^+^ Treg compartment.

**Figure 6:**
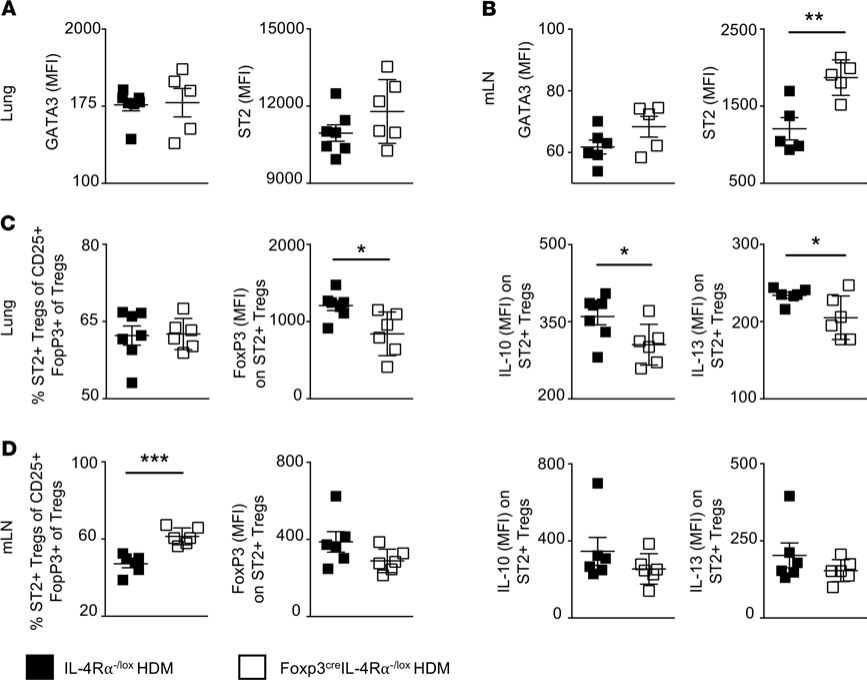
IL-4– and IL-13–responsive CD4^+^CD25^+^FoxP3^+^ Tregs regulate IL-10 production in the lung. Flow cytometry analysis of GATA3 and ST2 expression within CD4^+^CD25^+^Foxp3^+^ Tregs lung (**A**) and mLN (**B**) tissue. (**C**) Proportion of ST2^+^ Tregs in the lung and expression of FoxP3, IL-10, and IL-13. (**D**) Proportion of ST2^+^ Tregs in the mLN tissue and expression of FoxP3, IL-10, and IL-13. Mean ± SD of 1 representative experiment shown from 3 independent experiments (IL-4Rα^–/lox^ *n* = 6, Foxp3^cre^ IL-4Rα^–/lox^ *n* = 6). *P < 0.05, **P < 0.01. Mann-Whitney *U* test was performed.

Previously, disruption of Treg stability by proinflammatory cytokines in the lung has been shown to result in an unrestrained Th2 inflammatory response and a reprogramming of Tregs into Th2-like cells, thus exacerbating inflammation (18, 26). Therefore, we assessed the expression of GATA3 (a Th2 transcription factor) and ST2, an IL-33 receptor subunit, to identify a Th2-like reprogramming within FoxP3^+^ Tregs. We observed similar Gata3 expression in both mLN and lung tissue in FoxP3^+^ Tregs (Figure 6, A and B). However, a significant increase in ST2^+^ Tregs was observed in mLNs but not lungs of Foxp3^cre^ IL-4Rα^–/lox^ mice compared with IL-4Rα^–/lox^ littermate control mice (Figure 6, A and B). Although ST2 expression increased in mLN FoxP3^+^ Tregs of Foxp3^cre^ IL-4Rα^–/lox^ mice, this did not correlate with IL-13 expression (Figure 6B and Supplemental Figure 4B). It has recently been reported that ST2^+^ Tregs secrete IL-13 to reshape the myeloid compartment and control local inflammation after lung injury (42). Therefore, we further examined these ST2^+^ Tregs in the lung and observed impaired IL-13 production in Foxp3^cre^ IL-4Rα^–/lox^ mice compared with IL-4Rα^–/lox^ mice (Figure 6C and Supplemental Figure 4A). Additionally, a significant reduction was observed in Arg1^+^ myeloid cells (reparative myeloid cells) in Foxp3^cre^ IL-4Rα^–/lox^ mice compared with IL-4Rα^–/lox^ littermate control mice, indicating an altered myeloid compartment and reduced regulation of epithelial damage (Supplemental Figure 6, A and B) (42).

ST2^+^ Tregs are identified as highly activated, strongly suppressive cells, with a Th2 bias mainly in nonlymphoid tissues, and their suppressive capacity is mediated by IL-10 production (43). Lung ST2^+^ Tregs had significantly impaired FoxP3 expression and IL-10 secretion in Foxp3^cre^ IL-4Rα^–/lox^ mice compared with IL-4Rα^–/lox^ littermate control mice, confirming an altered regulatory function (Figure 6C and Supplemental Figure 5A). Interestingly, ST2^+^ Treg population in mLN tissue had comparable expression levels of FoxP3 and IL-10 between Foxp3^cre^ IL-4Rα^–/lox^ mice and IL-4Rα^–/lox^ mice, suggesting a maintained regulatory potency (Figure 6D and Supplemental Figure 5B). Here, we show an important function of IL-4Rα–responsive FoxP3^+^ Tregs in the stability of ST2^+^ Tregs, particularly in their suppressive function during HDM-induced allergic airway inflammation.

## Discussion

In this report, we show that lack of IL-4Rα signaling on Tregs, in the context of HDM-induced allergic asthma, results in uncontrolled lung pathology and airway hyperreactivity. We employed a murine model in which we deleted IL-4Rα on FoxP3^+^ Tregs. Unexpected findings were observed, in which deficiency in IL-4Rα on Tregs highlighted a context specificity in regulatory function. We elucidate that FoxP3, a marker of Treg function, was maintained in secondary lymphoid tissue, but was severely disrupted in the lung, a site of allergic inflammation. Interestingly, we did not observe hyperactivation of Th2 cells, but rather dysregulated type 2 innate responses driven mainly by epithelium-derived IL-33. Notably, IL-4Rα signaling on FoxP3^+^ Tregs was essential in the accumulation of lung ST2^+^ Tregs required for secreting suppressive IL-10 and IL-13, required for promoting reparative arginase-expressing myeloid cells. Our results highlight a complex function of IL-4Rα signaling on FoxP3^+^ Tregs in regulating airway hyperreactivity and lung pathology during HDM-induced allergic asthma.

A common role for Tregs is the control of the adaptive allergen-specific Th2 immune response (4, 17, 44). Moreover, deficiencies in Treg numbers or Treg trafficking in local tissue leads to an unrestrained allergen-specific Th2 immune cell response (4, 32, 45–47). We recently showed in a helminth model an exacerbated Th2 immune response in mice deficient in IL-4Rα on FoxP3^+^ Tregs (27). However, in HDM-induced allergic asthma we did not observe this unrestrained Th2 inflammatory response, but rather an innate type 2 response. Although T cells commonly contribute to the severity of asthma, they appeared to play a nonsignificant part in the exacerbation of airway hyperreactivity and remodeling in the absence of IL-4Rα signaling on Tregs. Pillemer and colleagues reported on the ability of Tregs to inhibit allergen sensitization and, consequently, HDM-induced airway inflammation regardless of their responsiveness to IL-4 (25). Our study also shows a maintained ability of T cells to produce proinflammatory Th2 cytokines (25). In the absence of IL-4Rα on FoxP3^+^ Tregs, allergen-specific cytokines reduced in the mLNs. However, these did not seem to originate from T cells. FoxP3 expression, which we used as a proxy for Treg suppressive ability, was maintained in mLNs, and we speculate it is a result of an increase in potent regulatory ST2-expressing Tregs with intact IL-10 secretion (43, 48). However, local lung tissue Tregs were severely disrupted in the absence of IL-4Rα on FoxP3^+^ Tregs, shown by reduced ability to secrete regulatory IL-10 and reduced levels of ST2^+^ Tregs. This is consistent with previous studies, in which a lack of ST2 on FoxP3^+^ Tregs resulted in reduced accumulation of intestinal Tregs and their ability to adapt to tissue microenvironment and suppress intestinal inflammation (49). Suppressive IL-10 secretion is an integral part of Treg suppressive ability (4, 13), and ST2^+^ Tregs also require IL-10 for their suppressive function (49). Our results were consistent with these findings and demonstrated reduced ability of both Tregs and CD4^+^ T cells to secrete IL-10 (13). Recently, ST2^+^ Tregs have been shown to be key in maintaining barrier immunity by secreting IL-13, which modulates the regulatory Arg1^+^ myeloid cells to repair epithelial damage (42). Our study shows that, in the absence of IL-4Rα signaling on FoxP3^+^ Tregs, there is impaired IL-13 secretion by ST2^+^ Tregs, which control epithelial repair.

Tregs have been shown to have a propensity to be hijacked and reprogrammed under the Th2 microenvironment where they lose their FoxP3 and gain Th2-associated transcriptional factors. In these settings, they lose their suppressive function and increase the pool of Th2 cells, which can contribute to disease pathogenesis (20, 24, 26). However, here we observed no gain in GATA3 expression upon loss of FoxP3 in the absence of IL-4Rα signaling on Tregs. Our studies partly contrast earlier findings, which showed a reprogramming of FoxP3^+^ Tregs into ex-FoxP3 Th2-like cells during HDM-induced allergic asthma (26). Interestingly, this reprogramming of FoxP3^+^ cells had a modest impact on FoxP3 in both lung and mLNs, but reduced BAL fluid eosinophilia, a phenotype we also observed in our model (data not shown). In addition, we observed no major changes in antigen-specific antibody production when IL-4Rα signaling on Tregs was disrupted, which validated previous findings that showed no altered antibody responses in the absence of IL-4Rα signaling on Tregs (26, 27). Our findings corroborate other studies in which IL-4–deficient Tregs demonstrated incompetency and inferior Treg-mediated immune suppression for both antigen-specific and nonspecific T cell activation (33).

Tregs show tissue specificity during inflammation, especially at mucosal sites, and often adapt to local environmental cues, which enhances their suppressive function (29, 30). IL-4 activation of FoxP3^+^ Tregs influences their ability to maintain barrier immunity through their regulatory function of ILC2 activation during allergic disease (11, 50). Here, we show an immunoregulatory role by IL-4Rα–responsive Tregs in modulating ILC2 expansion and function in vivo, revealing an important checkpoint for controlling ILC2-mediated type 2 immune responses. This marked ability of iTregs to regulate the communication between epithelial cells and ILC2s is critical in acute and chronic disease (51). The mechanism of modulating the innate cellular response involves restraining of epithelium-derived IL-33 secretion in lung tissue. This in turn blocked IL-33–induced ILC2 expansion, rich in IL-5 and IL-13 cytokines, which drive recruitment of eosinophils and activation of mast cells and basophils in the local tissue (52, 53). The crucial network of this interaction between epithelial cells and ILC2s generates sustained IL-33 production, which is key in the development of persistent/chronic asthma (51). Sustained barrier disruption induced by HDM proteolytic Derp-1 activity leads to increased IL-33 cytokine production (54, 55). We speculate that both IL-10^+^ Tregs and T cell–derived IL-10 may be critical in suppressing ILC2 activation (56). This idea would be consistent with previous studies in which Treg-derived IL-10 was able to suppress ILC2-driven papain-induced airway inflammation via an IL-33/mast cell axis. Additionally, it is likely that direct Treg-ILC2 contact via adhesion molecules such as ICOS–ICOS ligand exist, which have been shown to block ILC2 proliferation and type 2 cytokine secretion (11, 44).

This study highlights a complex scenario in the function of IL-4Rα–responsive Tregs, in which they act to suppress ILC2s through IL-10 production and control IL-33–mediated allergic inflammation. The local inflammatory environment is crucial in providing local cues that dictate Treg expansion and function. In this context, IL-4Rα–responsive Tregs show incredible specificity in the lung and direct suppression of lung ILC2s but not Th2 cells. We therefore provide evidence for the indispensable role of IL-4Rα signaling in maintaining Treg stability and IL-4–responsive Tregs in limiting IL-33–derived ILC2-driven AHR and airway inflammation in HDM-induced allergic asthma. Further studies are necessary to elucidate the exact mechanisms of epithelial immune modulation by Tregs and the lung-specific cues modulating Treg stability during type 2 airway immune responses. Additionally, our findings caution against the use of anti–IL-4Rα therapy, which may have undesirable interference in antigen-specific Treg-based tolerance (25), which we show as key in restraining IL-33–mediated allergic inflammation.

## Methods

### Mice.

Transgenic Foxp3^Cre^ mice (obtained from James Wing, Osaka University, Osaka, Japan) on BALB/c background were intercrossed for 2 generations with IL-4Rα^−/−^ BALB/c mice (57) to generate a Foxp3^Cre^ IL-4Rα^−/lox^ (27), and IL-4Rα^−/lox^ (58) hemizygotes on a BALB/c background were backcrossed up to 10 generations. All mice were used at 8–10 weeks of age. Mice were housed in independently ventilated cages under specific pathogen–free conditions in the University of Cape Town Animal Facility.

### HDM-induced allergic airway disease.

Mice were anesthetized with ketamine (Anaket-V; Centaur Labs) and xylazine (Rompun; Bayer) and sensitized intratracheally with 1 μg HDM (Stellergens Greer Laboratories) on day 0. Mice were i.n. challenged with 10 μg HDM under anesthesia on days 6, 7, 8, 9, and 10 as previously described (59). AHR was measured on day 14. After the procedure, mice were euthanized, and tissue samples were taken for analysis.

### AHR.

Airway resistance and elastance of the whole respiratory system (airways, lung, chest wall) after i.n. challenge was determined by forced oscillation measurements as described previously (60) with the Flexivent system (SCIREQ) by using the single-compartment (‘‘snapshot’’) perturbation. Measurements were carried out on mice with increasing doses of acetyl-β-methylcholine (MilliporeSigma) treatment. Differences in the dose-response curves were analyzed by repeated-measures ANOVA with Bonferroni’s posttest. Only mice with acceptable measurements for all doses (coefficient of determination > 0.90) were included in the analysis.

### Flow cytometry.

BAL fluid cells were obtained as previously described (61). Single-cell suspensions were prepared from lymph nodes in IMDM (Gibco) by passing them through a 100 μm filter. To obtain single-cell suspensions from lung tissues, a left lobe lung was digested for 1 hour at 37°C in RPMI (Gibco) containing 13 mg/mL DNase I (Roche) and 50 U/mL collagenase IV (Gibco) and passed through a 70 μm filter. IL-4Rα surface expression was detected on lymph node cells, lung cells, and BAL fluid cells by phycoerythrin (PE) anti-CD124 (IL-4Rα, M-1). Antibodies used in these experiments included (Table 1) PE (clone, E50-2440), anti-CD124 (IL-4Rα, clone, M-1), anti–IL-5 (clone, TRFK5), anti-Ki67 (clone, B56), anti-CD44 (clone, KM114), FITC-conjugated anti–Gr-1 (clone, RB6-8C5), CD45 (clone, 30-F11), IL-4 (clone, 11B11), PerCP Cy5.5-conjugated anti-Ly6C (clone, AL-21) and -CD45.1 (clone, A20), anti–IL-17 (clone, TC11-18H10), APC-conjugated anti-CD11c (clone, HL3) anti–IL-10 (clone, JES5-16E3), anti-FoxP3 (clone, MF23), V450-conjugated anti-CD11b (clone, M1/70), anti-CD62L (clone, MEL-14), Alexa Fluor 700–conjugated anti-CD3ε (clone, 145-2C11) anti–IFN-γ, V500–anti-CD4 (clone, RM4-5) and –anti-B220 (clone, RA3-6B2), APC-Cy7–conjugated anti-CD19 (clone, 1D3), anti-EpCAM (clone, G8.8), anti-CD8 (clone, 53-6.7), biotin-CD25 (clone, 7D4), and PerCP Cy5.5 Lineage antibody cocktail were purchased from BD Biosciences. PE-Cy7 anti-F4/80 (clone, BM8), anti–IL-13 (clone, eBio13A), Alexa Fluor 700–conjugated anti-MHC II (clone, M5/114), and LIVE/DEAD Fixable Yellow stain (Qdot 605 dead cell exclusion dye) were purchased from eBioscience. Arginase (Arg1), FITC–anti–SCA-1 (clone, D7), PE-Cy7 anti-GATA3 (clone, L50-823), and FITC–anti-T1/ST2 (clone, DJ8) were purchased from MD Bioproducts, MD Biosciences. PE–anti–IL-33 (clone, 396118) was purchased from Invitrogen. Biotin-labeled antibodies were detected by Texas red–conjugated PE (BD Biosciences). For staining, cells (1 × 10^6^) were labeled and washed in PBS, 3% FCS FACS buffer. For intracellular cytokine staining, cells were restimulated with PMA (MilliporeSigma) (50 ng/mL), ionomycin (MilliporeSigma) (250 ng/mL), and monensin (MilliporeSigma) (200 mM in IMDM/10% FCS) for 6 hours at 37°C, then fixed in 2% paraformaldehyde and permeabilized, and cytokine production was analyzed on an LSR Fortessa machine (BD Immunocytometry Systems). Data were analyzed using FlowJo software (Tree Star). All antibodies were from BD Pharmingen, BD Biosciences, unless otherwise noted.

### Histology.

Lungs were fixed in 4% formaldehyde/PBS and embedded in paraffin. Tissue sections were stained with periodic acid–Schiff for mucus secretion and with H&E for inflammation. Image analysis was performed on NIS Elements (Nikon Instruments). Mucus quantification was carried out using the automated NIS Elements software by defining regions of interest (ROIs), which are the individual bronchioles on cut lung sections to be analyzed for mucus staining, and using threshold quantification of the mucus stain in the specific ROIs in NIS Elements. Area of staining is defined as total area of mucus secretion per area of bronchiole epithelial lining. Lung sections from individual mice were assessed, and data from 3 experiments were pooled (*n* = 4–6 mice per experiment, 27–57 airway bronchioles analyzed per group).

### Antibody and cytokine ELISAs.

Antibody ELISAs were carried out as previously described (61) using 10 μg/mL HDM to coat for specific IgE and 5 μg/mL HDM to coat for specific IgGs. For in vitro cytokine production analysis, single-cell suspensions were prepared from mediastinal lymph nodes of HDM-treated and littermate control mice (61). Cells (2 × 10^5^ cells, in 200 μL) were incubated for 5 days in IMDM/10% FCS (Delta Bioproducts) in 96-well plates. Cells were either stimulated with HDM (30 μg/mL) or anti-CD3 (10 μg/mL), and supernatants were collected after a 5-day incubation period. BAL cells were isolated from BALF, lungs were homogenized, and supernatant was assessed for cytokine production after quantifying the protein concentration using a BCA kit (Thermo Fisher Scientific). IL-4, IL-5, IFN-γ, (BD Biosciences), IL-13, IL-33 (R&D Systems), IL-17, and IL-10 (BioLegend) concentrations were measured using ELISAs, according to the manufacturer’s protocol.

### Statistics.

Statistical significance was determined by nonparametric Mann-Whitney *U* test, 1-way ANOVA with Tukey’s test, or 2-way ANOVA with Bonferroni’s posttest for multiple comparisons using GraphPad Prism 6 software. Results are presented as mean ± SEM or mean ± SD. *P* values of less than 0.05 were considered significant.

### Study approval.

Animal procedures were performed according to strict recommendation by the South African Veterinary Council and were approved by the University of Cape Town Animal Ethics Committee (reference number 014/019 or 018/013).

## Author contributions

FB, FK, and SH conceived and supervised the study. JK, SH, and FK performed the experiments. JK, SH, and FB analyzed the data. JK, FB, and SH wrote the paper. All authors discussed the results and commented on the manuscript.

## Supplementary Material

supplemental data

## Figures and Tables

**Table 1:**
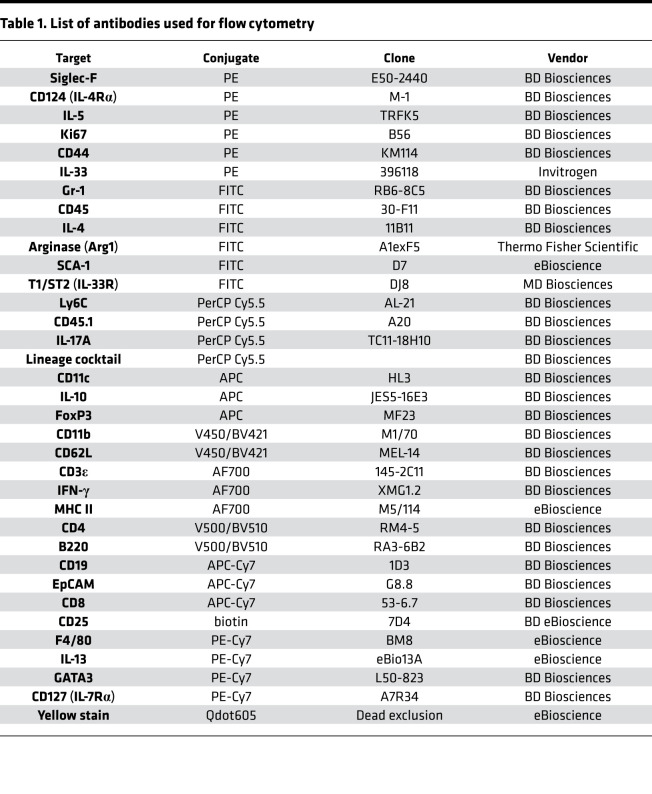
List of antibodies used for flow cytometry

## Version 1 09/15/2020

In-Press Preview

## Version 2 10/15/2020

Electronic publication

## References

[1] Eisner MD, Katz PP, Yelin EH, Henke J, Smith S, Blanc PD (1998). Assessment of asthma severity in adults with asthma treated by family practitioners, allergists, and pulmonologists. Med Care.

[2] Wing K, Sakaguchi S (2010). Regulatory T cells exert checks and balances on self tolerance and autoimmunity. Nat Immunol.

[3] Vignali DA, Collison LW, Workman CJ (2008). How regulatory T cells work. Nat Rev Immunol.

[4] Zhao ST, Wang CZ (2018). Regulatory T cells and asthma. J Zhejiang Univ Sci B.

[5] Rudensky AY (2011). Regulatory T cells and Foxp3. Immunol Rev.

[6] Josefowicz SZ (2012). Extrathymically generated regulatory T cells control mucosal TH2 inflammation. Nature.

[7] Barzaghi F, Passerini L, Bacchetta R (2012). Immune dysregulation, polyendocrinopathy, enteropathy, x-linked syndrome: a paradigm of immunodeficiency with autoimmunity. Front Immunol.

[8] Sakaguchi S, Yamaguchi T, Nomura T, Ono M (2008). Regulatory T cells and immune tolerance. Cell.

[9] Chinen T (2016). An essential role for the IL-2 receptor in T_reg_ cell function. Nat Immunol.

[10] Wing K (2008). CTLA-4 control over Foxp3+ regulatory T cell function. Science.

[11] Rigas D (2017). Type 2 innate lymphoid cell suppression by regulatory T cells attenuates airway hyperreactivity and requires inducible T-cell costimulator-inducible T-cell costimulator ligand interaction. J Allergy Clin Immunol.

[12] Akkaya B (2019). Regulatory T cells mediate specific suppression by depleting peptide-MHC class II from dendritic cells. Nat Immunol.

[13] Kearley J, Barker JE, Robinson DS, Lloyd CM (2005). Resolution of airway inflammation and hyperreactivity after in vivo transfer of CD4+CD25+ regulatory T cells is interleukin 10 dependent. J Exp Med.

[14] Soroosh P (2013). Lung-resident tissue macrophages generate Foxp3+ regulatory T cells and promote airway tolerance. J Exp Med.

[15] Georgiev P, Charbonnier LM, Chatila TA (2019). Regulatory T cells: the many faces of Foxp3. J Clin Immunol.

[16] Bacher P (2016). Regulatory T cell specificity directs tolerance versus allergy against aeroantigens in humans. Cell.

[17] Bacher P, Scheffold A (2018). The effect of regulatory T cells on tolerance to airborne allergens and allergen immunotherapy. J Allergy Clin Immunol.

[18] Chen CC, Kobayashi T, Iijima K, Hsu FC, Kita H (2017). IL-33 dysregulates regulatory T cells and impairs established immunologic tolerance in the lungs. J Allergy Clin Immunol.

[19] Wei J, Duramad O, Perng OA, Reiner SL, Liu YJ, Qin FX (2007). Antagonistic nature of T helper 1/2 developmental programs in opposing peripheral induction of Foxp3+ regulatory T cells. Proc Natl Acad Sci U S A.

[20] Noval Rivas M (2015). Regulatory T cell reprogramming toward a Th2-cell-like lineage impairs oral tolerance and promotes food allergy. Immunity.

[21] Burton OT (2014). Immunoglobulin E signal inhibition during allergen ingestion leads to reversal of established food allergy and induction of regulatory T cells. Immunity.

[22] Noval Rivas M, Burton OT, Oettgen HC, Chatila T (2016). IL-4 production by group 2 innate lymphoid cells promotes food allergy by blocking regulatory T-cell function. J Allergy Clin Immunol.

[23] Hershey GK, Friedrich MF, Esswein LA, Thomas ML, Chatila TA (1997). The association of atopy with a gain-of-function mutation in the alpha subunit of the interleukin-4 receptor. N Engl J Med.

[24] Massoud AH, Charbonnier LM, Lopez D, Pellegrini M, Phipatanakul W, Chatila TA (2016). An asthma-associated IL4R variant exacerbates airway inflammation by promoting conversion of regulatory T cells to TH17-like cells. Nat Med.

[25] Pillemer BB, Qi Z, Melgert B, Oriss TB, Ray P, Ray A (2009). STAT6 activation confers upon T helper cells resistance to suppression by regulatory T cells. J Immunol.

[26] Pelly VS (2017). Interleukin 4 promotes the development of ex-Foxp3 Th2 cells during immunity to intestinal helminths. J Exp Med.

[27] Abdel Aziz N, Nono JK, Mpotje T, Brombacher F (2018). The Foxp3+ regulatory T-cell population requires IL-4Rα signaling to control inflammation during helminth infections. PLoS Biol.

[28] Feng Y, Arvey A, Chinen T, van der Veeken J, Gasteiger G, Rudensky AY (2014). Control of the inheritance of regulatory T cell identity by a cis element in the Foxp3 locus. Cell.

[29] Wang Y, Su MA, Wan YY (2011). An essential role of the transcription factor GATA-3 for the function of regulatory T cells. Immunity.

[30] Zheng Y (2009). Regulatory T-cell suppressor program co-opts transcription factor IRF4 to control T(H)2 responses. Nature.

[31] Lin W (2007). Regulatory T cell development in the absence of functional Foxp3. Nat Immunol.

[32] Dorsey NJ, Chapoval SP, Smith EP, Skupsky J, Scott DW, Keegan AD (2013). STAT6 controls the number of regulatory T cells in vivo, thereby regulating allergic lung inflammation. J Immunol.

[33] Yang WC (2017). Interleukin-4 supports the suppressive immune responses elicted by regulatory T cells. Front Immunol.

[34] Wills-Karp M (1998). Interleukin-13: central mediator of allergic asthma. Science.

[35] Tibbitt CA (2019). Single-cell RNA sequencing of the T helper cell response to house dust mites defines a distinct gene expression signature in airway Th2 cells. Immunity.

[36] Halim TY, Krauss RH, Sun AC, Takei F (2012). Lung natural helper cells are a critical source of Th2 cell-type cytokines in protease allergen-induced airway inflammation. Immunity.

[37] Klein Wolterink RG (2012). Pulmonary innate lymphoid cells are major producers of IL-5 and IL-13 in murine models of allergic asthma. Eur J Immunol.

[38] Barlow JL (2012). Innate IL-13-producing nuocytes arise during allergic lung inflammation and contribute to airways hyperreactivity. J Allergy Clin Immunol.

[39] Oboki K (2010). IL-33 is a crucial amplifier of innate rather than acquired immunity. Proc Natl Acad Sci U S A.

[40] Kubo M (2017). Innate and adaptive type 2 immunity in lung allergic inflammation. Immunol Rev.

[41] Cayrol C, Girard JP (2018). Interleukin-33 (IL-33): A nuclear cytokine from the IL-1 family. Immunol Rev.

[42] Liu Q (2019). IL-33-mediated IL-13 secretion by ST2+ Tregs controls inflammation after lung injury. JCI Insight.

[43] Siede J (2016). IL-33 Receptor-expressing regulatory T cells are highly activated, Th2 biased and suppress CD4 T cell proliferation through IL-10 and TGFβ released. PLoS One.

[44] Hurrell BP, Shafiei Jahani P, Akbari O (2018). Social networking of group two innate lymphoid cells in allergy and asthma. Front Immunol.

[45] Mock JR (2014). Foxp3+ regulatory T cells promote lung epithelial proliferation. Mucosal Immunol.

[46] Umetsu DT, Dekruyff RH (2006). Immune dysregulation in asthma. Curr Opin Immunol.

[47] Curotto de Lafaille MA, Kutchukhidze N, Shen S, Ding Y, Yee H, Lafaille JJ (2008). Adaptive Foxp3+ regulatory T cell-dependent and -independent control of allergic inflammation. Immunity.

[48] Akdis CA, Akdis M (2014). Mechanisms of immune tolerance to allergens: role of IL-10 and Tregs. J Clin Invest.

[49] Schiering C (2014). The alarmin IL-33 promotes regulatory T-cell function in the intestine. Nature.

[50] Redpath SA (2013). ICOS controls Foxp3(+) regulatory T-cell expansion, maintenance and IL-10 production during helminth infection. Eur J Immunol.

[51] Christianson CA (2015). Persistence of asthma requires multiple feedback circuits involving type 2 innate lymphoid cells and IL-33. J Allergy Clin Immunol.

[52] Nakae S, Morita H, Ohno T, Arae K, Matsumoto K, Saito H (2013). Role of interleukin-33 in innate-type immune cells in allergy. Allergol Int.

[53] Wawrzyniak P (2017). Regulation of bronchial epithelial barrier integrity by type 2 cytokines and histone deacetylases in asthmatic patients. J Allergy Clin Immunol.

[54] Martín-Orozco E, Norte-Muñoz M, Martínez-García J (2017). Regulatory T cells in allergy and asthma. Front Pediatr.

[55] Cayrol C, Girard JP (2014). IL-33: an alarmin cytokine with crucial roles in innate immunity, inflammation and allergy. Curr Opin Immunol.

[56] Morita H (2015). An interleukin-33-mast cell -interleukin-2 axis suppresses papain-induced allergic inflammation by promoting regulatory T cell numbers. Immunity.

[57] Mohrs M, Ledermann B, Köhler G, Dorfmüller A, Gessner A, Brombacher F (1999). Differences between IL-4- and IL-4 receptor alpha-deficient mice in chronic leishmaniasis reveal a protective role for IL-13 receptor signaling. J Immunol.

[58] Herbert DR (2004). Alternative macrophage activation is essential for survival during schistosomiasis and downmodulates T helper 1 responses and immunopathology. Immunity.

[59] Debeuf N, Haspeslagh E, van Helden M, Hammad H, Lambrecht BN. Mouse models of asthma. In: *Current Protocols in Mouse Biology*. John Wiley & Sons, Inc.; 2016:169–184. 10.1002/cpmo.4 27248433

[60] Nieuwenhuizen NE (2012). Allergic airway disease is unaffected by the absence of IL-4Rα-dependent alternatively activated macrophages. J Allergy Clin Immunol.

[61] Kirstein F (2010). Expression of IL-4 receptor alpha on smooth muscle cells is not necessary for development of experimental allergic asthma. J Allergy Clin Immunol.

