# Supplementary material for: IL-4Rα signaling in CD4+CD25+FoxP3+ T regulatory cells restrains airway inflammation via limiting local tissue IL-33
Source: JCI Insight. 2020 Oct 15;5(20):e136206. doi: 10.1172/jci.insight.136206 (PMC7605533; doi:10.1172/jci.insight.136206)
Supplement: supplemental data [file jciinsight-5-136206-s050.pdf]

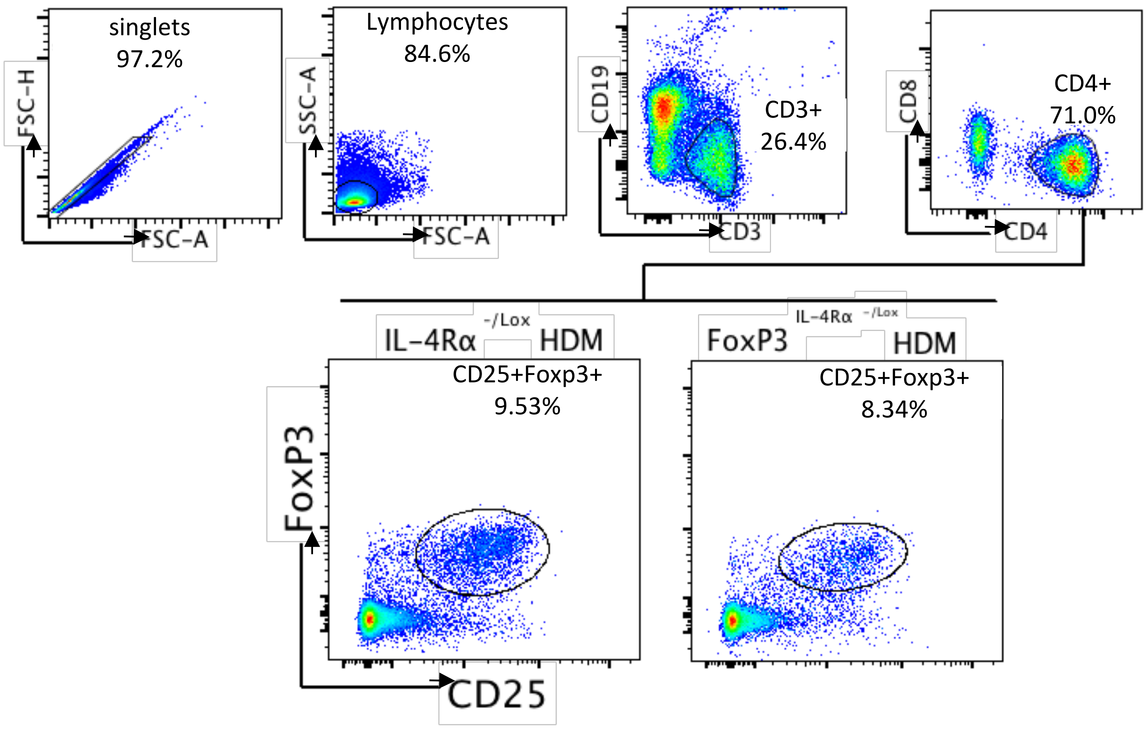


**Supplemental Figure 1. Gating strategy for CD4$^{+}$ CD25$^{+}$ FoxP3$^{+}$ T regulatory cells in both the mLN and lung tissue.**

We counted live cells by trypan blue and excluded dead cells by 7AAD or Qdot605 live/dead stain marker. We excluded doubles and gated on lymphocytes. To get to T reg cells, we surface stained with CD19-CD3+, followed by CD4+CD8-CD25+. To detect Foxp3, we fixed the cells and permeabilised with eBioscience Foxp3 kit before staining for intracellular transcriptional factor Foxp3.

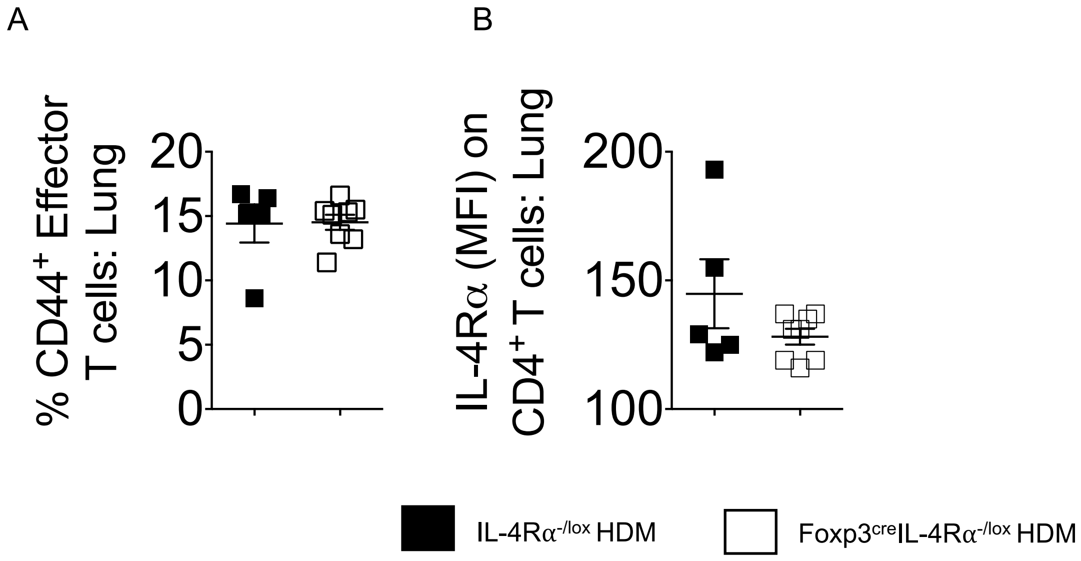


**Supplemental Figure 2. Lung CD4 T cells are not impacted by lack of IL-4Rα signalling on T regs.**

(**A**) Effector phenotype of CD4 T cells ($CD3^+CD4^+CD44^+CD62L^-$)

(**B**) Expression of IL-4Rα in lung CD4 T cells ($CD3^+CD4^+CD44^+CD62L^-$)

Related to Figure 2

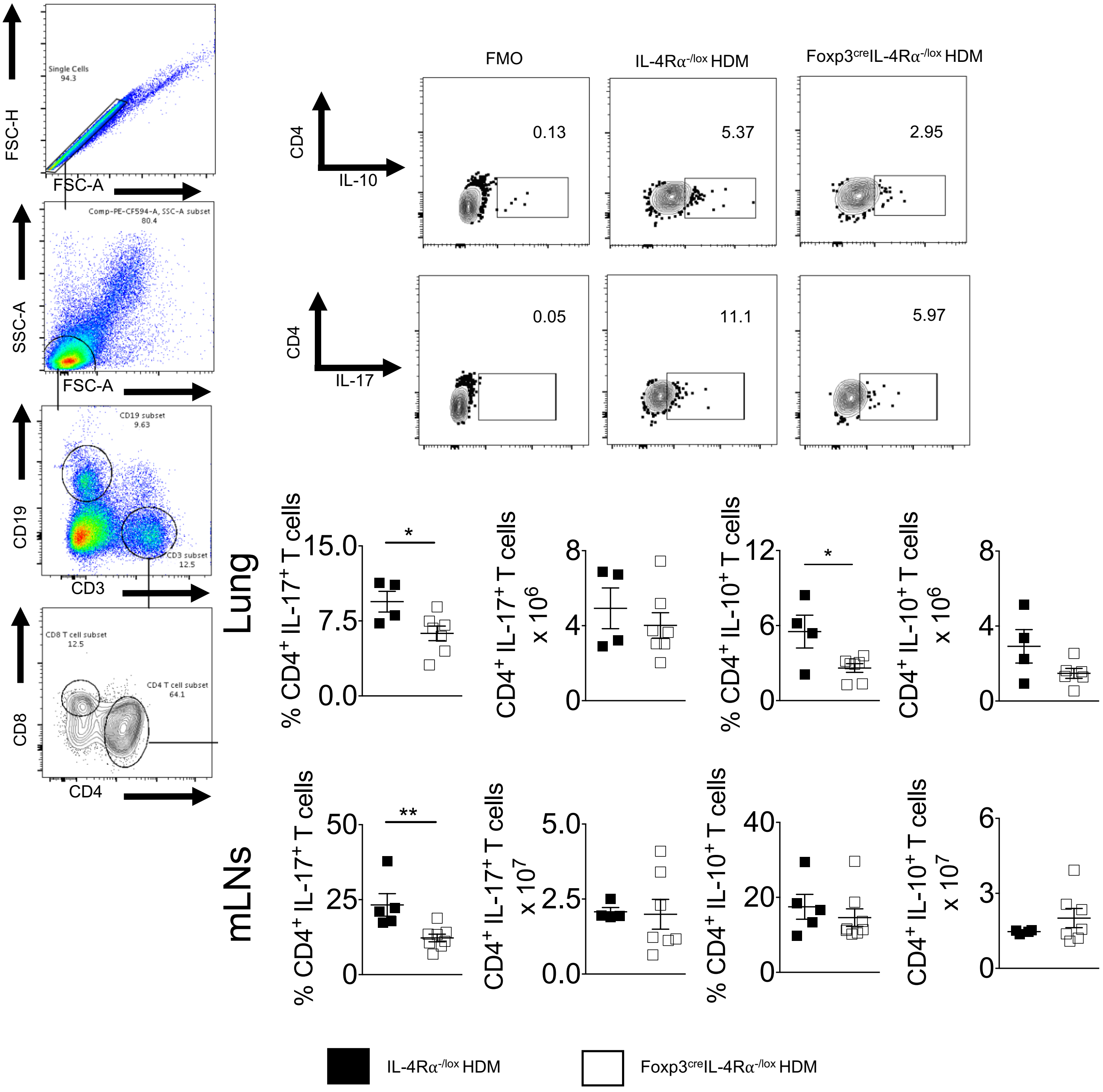


**Supplemental Figure 3. Flow cytometry plots and gating strategy for CD4$^+$ T cells producing intracellular IL-10 and IL-17 after 5 hr stimulation with PMA/ionomycin and monensin**. Cellular numbers of cytokine producing CD4$^+$ T cells calculated based on percentages.

Data shown is mean ± SDs 1 representative experiment of 3 independent experiments (n=4-7). Significant differences between groups are represented as *p < .05. Part of Figure 4.

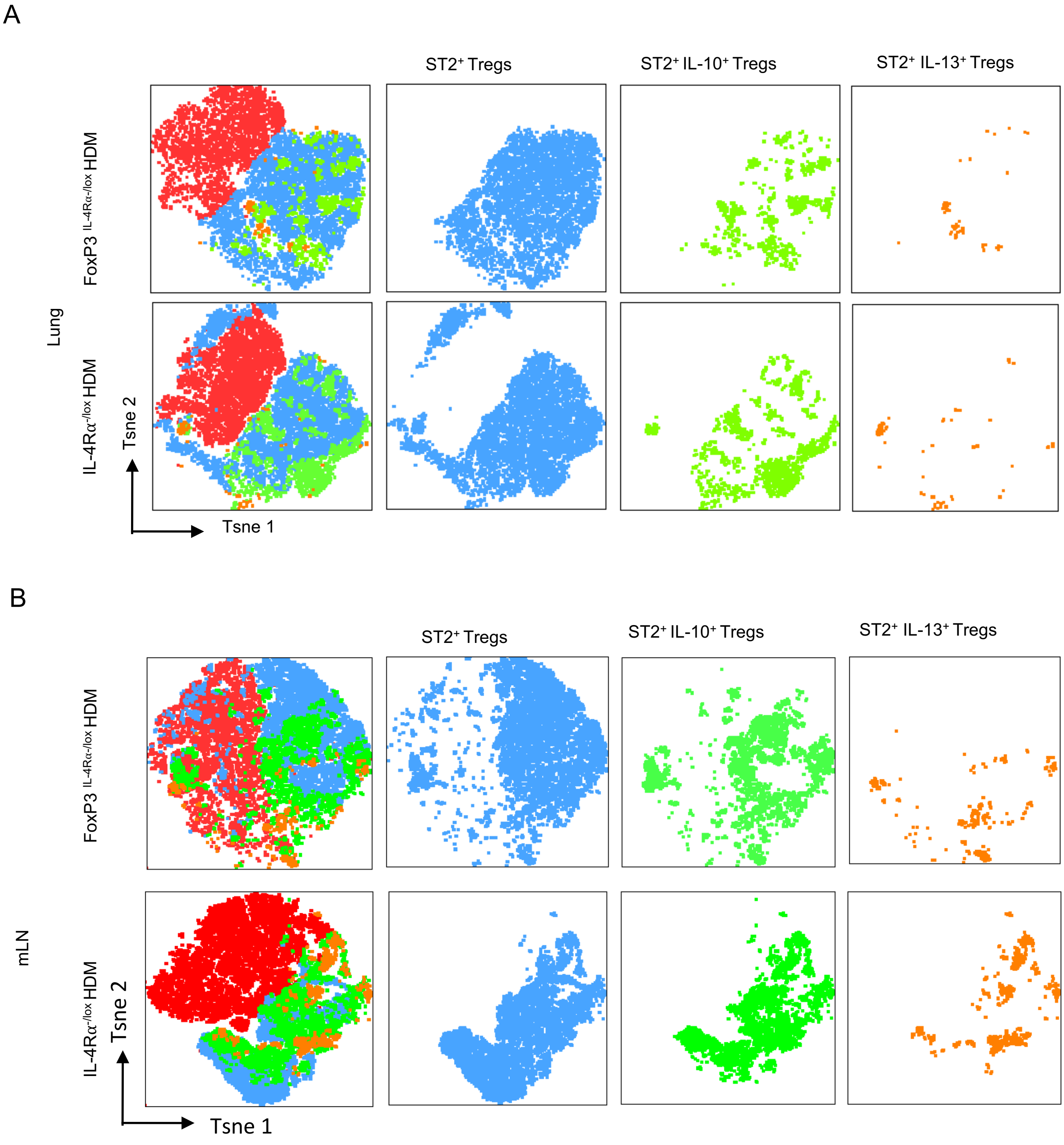


**Supplemental Figure 4. tSNE dimensional analysis for the CD25$^+$ FoxP3$^+$ T regulatory cell compartment**

(**A**) Lung and

(**B**) mLN.

Related to Figure 6

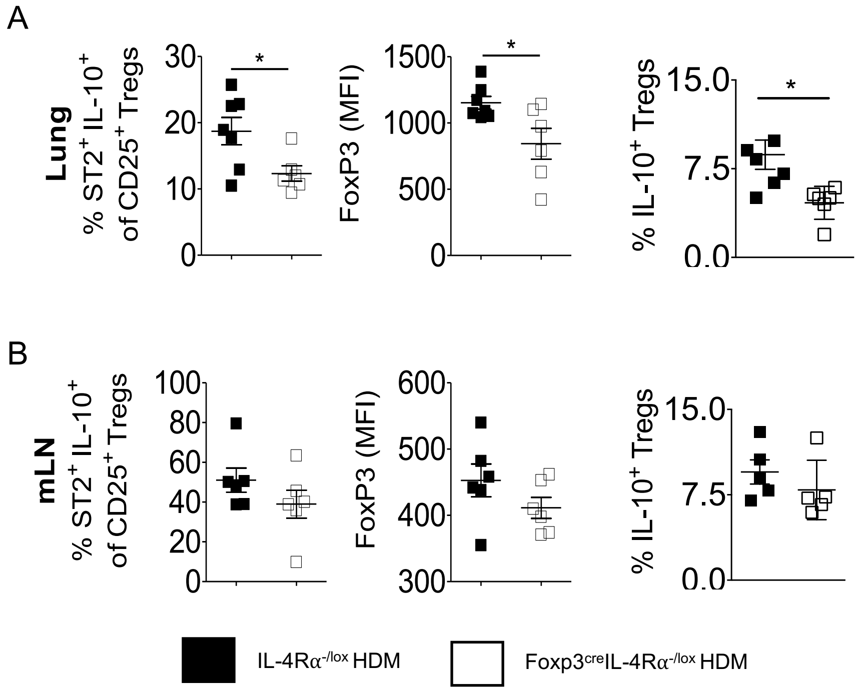


**Supplemental Figure 5:** Modulation of suppressive Treg compartment in the absence of IL-4Rα signaling on T reg cells .

(**A**) % of $ST2^{+}IL\text{-}10^{+}$ $CD25^{+}$ Treg cells (left panel), Foxp3 expression within the $ST2^{+}IL\text{-}10^{+}$ $CD25^{+}$ Treg cells (middle panel), % IL-10 expression within $CD25^{+}ST2^{+}$ Treg cells (right panel) in the lung.

(**B**) % of $ST2^{+}IL\text{-}10^{+}$ $CD25^{+}$ Treg cells (left panel), Foxp3 expression within the $ST2^{+}IL\text{-}10^{+}$ $CD25^{+}$ Treg cells (middle panel), % IL-10 expression within $CD25^{+}ST2^{+}$ Treg cells (right panel) in the mediastinal lymph nodes.

Data shows mean ±SDs from 1 representative experiment of 3 independent experiments carried out (n= 5-8 per group). Significant differences are described as: *$p < .05$
MFI, Median fluorescence intensity; mLNs, mediastinal lymph nodes
Figure related to Figure 6.

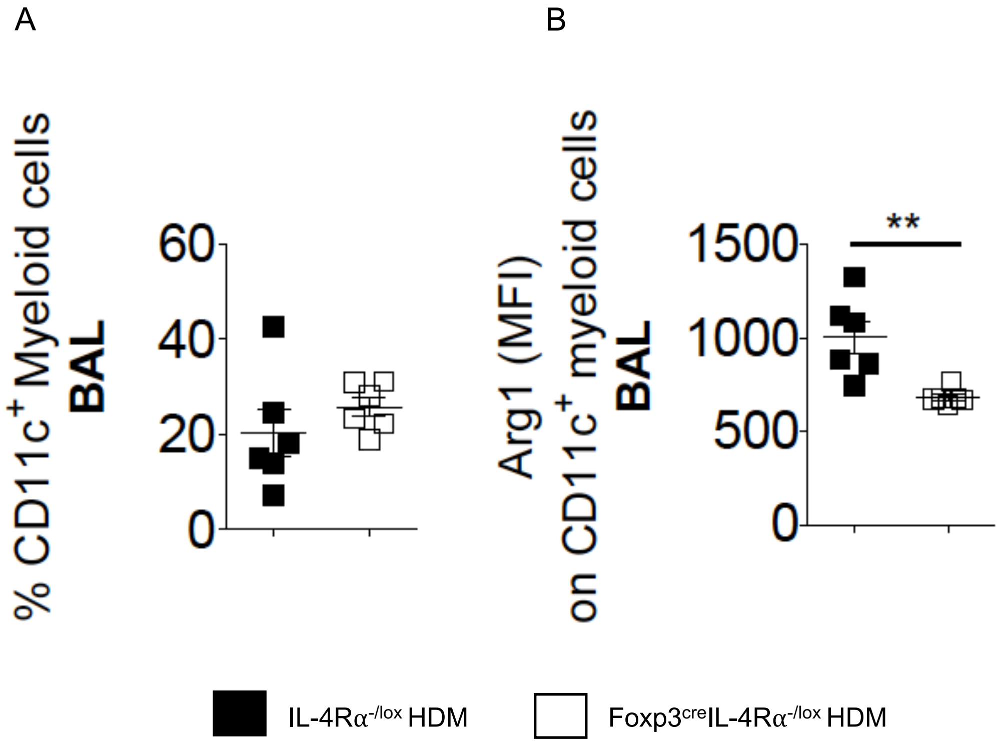


**Supplemental Figure 6:** CD11c+ Myeloid cells are altered in the absence of IL-4Rα signalling in T reg cells during HDM induced allergic asthma.

(**A**) Proportions of CD11c+ myeloid cells in BAL.

(**B**) Expression of arginase 1 on CD11c+ myeloid cells in BAL fluid
